# Impact of intelligence on social cognition in mentally disordered offenders: preliminary evidence in schizophrenia and personality disorders

**DOI:** 10.3389/fpsyg.2026.1731232

**Published:** 2026-03-12

**Authors:** Kyriakos Ioannidis, Lena Magnenat, François R. Herrmann, Younes Boulaguiem, Laila Espinosa, Panteleimon Giannakopoulos, Kerstin Weber

**Affiliations:** 1 Division of Institutional Measures, Medical Direction, Geneva University Hospitals, Geneva, Switzerland; 2 Department of Rehabilitation and Geriatrics, Geneva University Hospitals and University of Geneva, Thônex, Switzerland; 3 Department of Psychiatry, University of Geneva, Geneva, Switzerland; 4 Health Office, Department of Health and Transport, State of Geneva, Geneva, Switzerland

**Keywords:** emotional intelligence, forensic psychiatry, offenders, social cognition, theory of mind

## Abstract

**Introduction:**

Social cognition (SC) allows individuals to perceive, interpret, and react to intentions and behaviors of others. Forensic psychiatry patients show impaired SC, leading to emergence of violent behaviors. This impairment may differ according to patients’ intelligence quotient and psychiatric diagnosis (schizophrenia spectrum vs. personality disorders).

**Aims:**

The study investigated emotional SC and cognitive SC in male forensic inpatients, exploring demographic, criminological and clinical determinants. *Methods*: We retrospectively retrieved and analyzed data in a preliminary sample of 75 offenders following court-ordered psychiatric treatment in Switzerland. Emotional SC was assessed using a facial emotions’ recognition task and cognitive SC was assessed with theory of mind comics picture stories. Multiple logistic regression models were applied to identify protective and risk factors of cognitive SC and emotional SC.

**Results:**

39.2% of the offenders presented cognitive SC and 25.3% emotional SC impairment. Higher intelligence quotient was the unique 10-fold protective factor for cognitive SC, independently of psychiatric diagnoses and criminal history. In contrast, for emotional SC, average intelligence and PD disorder acted as protective factors compared to schizophrenia and intellectual deficiency, while foreign nationality acted as risk factor. Notably, neither recidivism nor the violent or multiple nature of crimes were related to SC.

**Discussion:**

Clinicians should consider patients’ level of intelligence and include differential emotional and cognitive SC assessments, when referring mentally disordered offenders for SC training to improve their prosocial skills.

## Introduction

Social cognition (SC), is defined as a complex set of mental abilities underlying social stimulus perception, processing, interpretation, and response. Together, these abilities support the development of adequate social competence and adaptation (Frith and Frith, 2007; Pinkham et al., 2014; Beaudoin and Beauchamp, 2020). Schurz et al. (2021) proposed a multilevel model of hierarchical structure of SC, where higher levels describe broader and more abstract neurocognitive functions (predominantly cognitive processes), whereas lower ones explain how these functions manifests in a concrete context and for a particular problem (more affective processes). Although there is a lack of agreement regarding concepts and taxonomy used to study social cognition, two terms are of central importance. Empathy, generally referred to as affective route for understanding others, is the ability to interpret other people’s internal states by analyzing external cues, particularly facial expressions (Decety and Jackson, 2004; Pinkham et al., 2014). Theory of Mind (ToM) or mentalizing, referring to the cognitive representations of others’ mental states, includes the ability to infer the mental state of others or take their perspective, such as beliefs and intentions (Decety and Jackson, 2004; Pinkham et al., 2014). For the ease of understanding, in this paper, we chose the terms of emotional SC to refer to facial affect recognition, and of cognitive SC to refer to the Theory of Mind.

Social cognition abilities have been positively related to general cognitive skills (intelligence quotient) both in healthy development (Conte et al., 2018a; Grazzani et al., 2018) and in children with intellectual disabilities (Charman and Campbell, 2002; Thirion-Marissiaux and Nader-Grosbois, 2008). During normal development, children acquire competence in social cognition as their verbal and non-verbal cognitive abilities increase (Wellman and Liu, 2004; Conte et al., 2018b; Schultz et al., 2018).

SC has been positively associated with the tendency to be prosocial, trustworthy, altruistic, or cooperative (Nettle and Liddle, 2008; Ferguson and Austin, 2010; Blain et al., 2021). It may be affected across a range of psychiatric disorders and, in particular, schizophrenia spectrum disorders (SSD) and Cluster B externalizing personality disorders (PD; Wilson et al., 2017; Jeandarme et al., 2022). Patients with SSD display marked impairments in processing social information, which can result in misinterpretations of the other’s intents, social withdrawal, and impaired daily functioning (Green et al., 2015). Both facial affect recognition and ToM have been consistently found to be impaired in SSD patients (Pinkham et al., 2014; Green et al., 2015). More than 70–75% of the schizophrenia patients present SC impairment, varying from mild (40%) to severe severity (32%; Hajdúk et al., 2018; Vaskinn and Horan, 2020). Although the prevalence rates of SC deficits in social cognition in Cluster B disorders (regrouping narcissistic, antisocial, and histrionic personalities) are quite variable, two meta-analyses focusing on borderline personality disorder showed that patients significantly underperformed healty controls in overall ToM, mental state reasoning, and cognitive ToM, but had no deficits in mental state decoding (Németh et al., 2018). Patients with borderline personality disorders are impaired in ToM capacities and hypersensitive to social threat and negative stimuli (Bora, 2021). Patients with antisocial personality disorders also show deficits in the recognition of negative emotional facial expressions (especially sadness and fear), although they may be good at perceiving others’ intentions and exhibit intact ToM (Marsh and Blair, 2008; Decety and Moriguchi, 2007). Their lack of empathy could be related to disrupted affective processing rather than an inability, for instance, to adopt the perspective of others.

SC is related to both psychiatric disorders and violent criminal behavior. Forensic researchers distinguish between affective reactive violence, which is a response to physical or verbal aggression initiated by others, that is often uncontrolled and emotionally charged, from a predatory cold-blooded aggression, which is a controlled, purposeful aggression that is used to achieve a desired goal (Dodge et al., 1997). The first type of violence may be associated with both cognitive and emotional SC impairment, whereas the second one reveals emotional SC impairment (Decety and Moriguchi, 2007). Some authors find that in offenders both components of SC are involved in the emergence of violent behaviors (Decety and Cowell, 2015). Violent offenders show deficits in facial affect recognition of fear and anger, alongside misjudgment of neutral expressions as indicative of fear or anger (Bulgari et al., 2020), but also reduced ability to infer the mental states of others, as revealed by impaired theory of mind tasks (Green et al., 2015; Friestad and Vaskinn, 2021). Criminal recidivists show impaired ToM, particularly in decoding intentionality, independently of offense type (Opriș et al., 2025). Being able to identify another person’s facial expression represents the first stage in the empathy unfolding process (Friestad and Vaskinn, 2021). Inability to correctly identify facial emotional cues could lead to a misunderstanding of social scenarios and other’s intents. This in turn may hinder the recognition of signs of distress in others, consequently eliminating the usual restraint that they provoke against violent behavior (Tasios et al., 2024). In contrast, other studies found that aggressive offenders show reduced empathic responses to emotional videos of others’ suffering, while Theory of Mind performance remains intact. These evidences stress the importance of distinguishing between affective and cognitive deficits in social cognition for understanding aggressive behavior (Winter et al., 2017).

SC may be particularly impaired in violent offenders *with* mental disorders. Indeed, most forensic inpatients suffer from schizophrenia spectrum disorders and Cluster B personality disorders (Margari et al., 2024). Incarcerated antisocial violent offenders show consistent hostile attribution bias toward ambiguous facial cues, that favor criminal recidivism (Schönenberg and Jusyte, 2014; Wegrzyn et al., 2017). Severe crime offenders with schizophrenia, who commit homicide, display emotion perception deficits and a tendency to under-mentalize compared to healthy controls (Engelstad et al., 2019). However, facial emotion recognition deficit may also exist in schizophrenics, independently of their violent or non-violent behavior, supporting the idea that SC impairment is a trait feature of the illness (Demirbuga et al., 2013; Battaglia et al., 2022; Tasios et al., 2024).

SC deficits are associated with increased occurrence of violent behaviors among SSD and PD patients (Herpertz and Bertsch, 2014; Pinkham et al., 2014), yet these deficits may differ according to both diagnostic groups. Some authors found that SSD patients perform worse than PD patients in theory of mind tasks (Murphy, 2006). Antisocial PD patients show reduced responsivity to emotions of others while being able to recognize and label others’ emotions, whereas schizophrenia show heightened reactivity to emotions of other sand a reduced ability to label others’ emotions or mental states (Schiffer et al., 2017). However, other studies found no differences in SC performances between the two patient groups. Patients with antisocial personality disorder and violent psychotic offenders showed no remarkable differences in affective or cognitive empathy tests between the two groups, also both patient groups presented deficits in empathy and theory of mind when compared to controls (Tasios et al., 2024).

One possible explanation for these discrepancies may reside to the general level of intellectual functioning, verbal memory, and cognitive flexibility (Murphy, 1998). Contrary to PD patients, SSD patients may be impaired in how and when to apply strategic social reasoning. Theory of mind deficits in SSD may thus be related to general intelligence (IQ) impairments, rather than reflecting a genuine compromised mental state attribution (Brüne, 2003). Another study showed that social cognition has a direct effect on violence in SSD independently of cognitive performance (O’Reilly et al., 2015). Besides, age, gender, and education are known to impact measures of cognitive and emotional SC. In a large international study across 12 countries, differences between countries accounted for more than 20% of the variance on both measures (Quesque et al., 2022). Studies also revealed cultural bias, due to an own-nationality advantage, individuals score better at recognizing facial expressions of basic emotions posed by individuals from their country than those of other-nationality members (Yan et al., 2016). Since Geneva is a very cosmopolitan city, we took this variable into account.

The aims of the present study were a. to analyze the patterns of emotional as well as cognitive SC in male forensic SSD and PD psychiatric inpatients, who had been sentenced to court-ordered treatment after repeatedly committing violent or non-violent offenses, and b. to explore the demographic, criminological and clinical predictors of SC deficits in the present sample. We expected emotional SC to be impaired in both patient groups, and cognitive SC to be impaired only in the SSD patient group. We hypothesized a lower intelligence quotient and a more severe history of violence (multiple offenses) to be associated with lower SC performance in both groups.

## Materials and methods

### Clinical assessment procedures

The retrospective observational design is based on a range of data routinely collected in a prison-based forensic inpatient psychiatric clinic in Geneva (Switzerland), offering court-ordered treatment for mentally disordered offenders. All inpatients followed the same court-ordered treatment in the same forensic psychiatry clinic, including psychotropic medication as well as psychotherapy individual and group sessions according to a forensic therapeutic community approach, that has been previously described (Weber et al., 2023). Demographics, past criminal history, and diagnosis as defined by the court-ordered expert assessment, are systematically recorded at admission to the clinic by the psychiatrist in charge. Psychiatric diagnoses are further confirmed in two independent clinical interviews of two fully trained psychiatrists, who are blind to each other and the medical file, according to ICD-10 criteria during the initial 3 months of inpatient treatment. Indeed, detailed diagnostic assessment plays a crucial role in forensic psychiatry and treatment outcome prognosis. Forensic-psychiatric institutions are high-cost, low volume services which pose significant ethical problems since the length of stays are often long and even indefinite. Diagnosis consensus is based on the majority (two concordant diagnoses out of 3) and solved by subsequent additional psychological assessment in case of majority lacking.

Neuropsychological assessment is performed within 6 months of the patient’s admission once clinically stable, and on the psychiatrist’s request whenever the medical examination reveals a suspicion of cognitive deficits. The neuropsychological assessment, performed by the same clinical neuropsychologist, is extensive and hypothesis-driven according to the psychiatrist’s clinical reasoning. However, given the forensic context, it routinely includes social cognition assessment and/or general intelligence (IQ) assessment with the measures detailed below. For social cognition, the GeSOCS vs. Sarfati task is selected by the neuropsychologist based on the patient’s educational level.

### Research data extraction and study sample

We considered the entire cohort of 125 SSD and Cluster B personality disorder (PD) patients who had been referred for a neuropsychological assessment between 2014 and 2024. To create a retrospective database, ethics approval was obtained in 2022. Data extraction was performed in 2025 by two senior legal psychologist and psychiatrist, trained in swiss forensic law procedures, and not involved in the care programs of the mentally disordered offenders. They conducted psychiatric case file reviews to extract demographic characteristics (age, civil and educational status, nationality), intelligence (intellectual quotient, IQ) and social cognition assessments. Legal case files were conducted to extract criminal history (nature and diversity of offenses, recidivism), as well as psychiatric diagnosis as documented by expert assessments, which are part of each legal investigation according to the Swiss criminal procedure.

**Figure 1:**
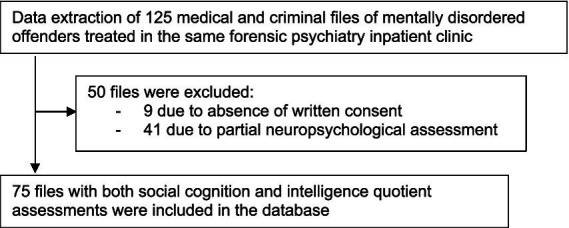
Study sample selection.

Among the 125 patients, 9 patients did not give their written consent for empirical data extraction. Among the 116 remaining patients, the data of 75 patients with both social cognition and intelligence quotient assessments were extracted and included in the database of the study, the data of the remaining 41 patients were not extracted because of non-standard neuropsychological assessment (Figure 1). Homogeneity of this environmental study sample is guaranteed by the fact that all patients are treated in the same and only adult Swiss forensic psychiatry clinic for the French and Italian speaking parts of Switzerland. All patients are submitted to the same court-ordered treatment to reduce the risk of violence and crime recidivism, based on the same forensic therapeutic community approach. Risk reduction - rather than healing from mental illness - is the core marker of treatment progress and the criterion to decide discharge from the forensic clinic. The 75 patients were divided into two broad SSD and PD categories, by analogy to previous studies and the two most frequent sources of interpersonal violence: psychotic loss of touch with reality on one hand and externalizing PD Cluster B dramatic, erratic, and emotional behaviors on the other hand. The SSD group included 51 inpatients with paranoid schizophrenia (F20.0), undifferentiated schizophrenia (F20.3), and schizotypal disorder (F21). The Cluster B personality disorder group included 24 inpatients with antisocial (F60.2), borderline (F60.3) and narcissistic personality disorder (F60.81).

Ethical approval for this study was obtained by the local Ethic Committee (project ID 2022-00739). Written informed consent for participation was obtained from participants still under treatment at the time of the study. It was not required for participants already discharged in accordance with the national legislation and institutional requirements.

### Measures

#### Social cognition assessment

Emotional SC was assessed by a facial emotions’ recognition test (Social Cognition & Emotional Assessment, Mini-SEA; Bertoux, 2014), validated in a French-speaking population. The test includes 35 pictures with 5 different faces representing 7 different emotions: joy, surprise, disgust, sadness, anger, fear, or neutrality. For each face, the subject must specify in less than 12 s which emotion is depicted. The seven choices of emotions are written below every picture. According to the scoring procedure, each correctly coded emotion equals 1 point for a total of 35 points. As part of a larger assessment, the total score is converted (total x 15/35) into a 15 points-score. Higher scores indicate better performance. Cut-off scores for impaired emotional SC was <10.85 on the facial emotion recognition test.

Cognitive SC was assessed with Theory of Mind comics picture stories (cartoons) representing false beliefs (e.g., A man is sitting on a bench with his dog beside him and he is preparing to eat a sandwich; while he is throwing the paper packaging, a bird steals the sandwich; on seeing the sandwich missing, the man scolds the dog. Question: why does the man scold his dog?). The patient is invited to carefully look at all the pictures and try to understand what is happening. Afterward, he is asked questions about the story and explicitly required to infer the characters’ mental state, meaning he is invited to answer questions based on what the characters are thinking, feeling, or intending, not just on what he can see. (e.g., beliefs: “What does X think is happening?”, intentions: “Why did X do that?” emotions: “How does X feel in this situation?”). Adapted to patient’s level of education, the subtest of the Geneva Social Cognition Scale (GeSOCS; Martory et al., 2015) or the Intention inference task of Sarfati et al. (1997) (validated in French language in Paris) were used. Subjects are asked to infer the character’s intention in specific contexts in short comic strips, each containing a target mental state. Understanding of the character’s intention depends on the interpretation of the character’s behavior in the comic strip. One answer card is a logical conclusion which matched the character’s intention, and two answer cards are distracters and have no logical link with the intentional context of the scenario. (e.g., A man is standing near a café table where another person has left a bag. The man looks at the bag, then around him. In the final picture, the man picks up the bag and starts walking. Question to the participant: “Why did the man pick up the bag?” The four response options: A. He wanted to steal the bag, B. He believed the bag belonged to him, C. He intended to return the bag to its owner, D. He wanted to examine what was inside.) Regarding the scoring, the GeSOCS inference task includes 5 comics strips, each rated 4 points (2 points for inference + 2 points for control question) added up for a total out of 20 points. The Sarfati inference task includes 30 comic strips, and each correct answer is rated 1 point, added up for a total out of 30 points. Higher scores indicate better performance.

We created a binary SC outcome variable (1 = intact, 0 = impaired social cognition) to allow for score comparison despite the different score ranges of each ToM measure. The cut-off scores to split patients’ results were those defined *a priori* for each measure as clinically significant cut-off scores to detect impairment. Cut-off scores for impaired cognitive SC were < 21.75 for the Intention interference task and < 18 for GeSOCS.

#### Assessment of determinants

To assess the general level of cognitive abilities, the Weschler intelligence scale, 4th edition (WAIS-IV), was extracted from the patient’s medical file. The prison neuropsychologist had applied the standard procedure of the French version paper-pencil battery in each inpatient, assessing the full 10 core subtests in a face-to-face interview. We divided the intelligence quotient (IQ) into three sub-groups: IQ < 70, 70 < IQ < 80, IQ > 80. Socio-demographic variables extracted from the file included age (in years), nationality (binary; 0 = foreigner vs. 1 = swiss nationality), marital status, as well as level of education (number of years of school, school drop-out). Extracted criminal offense characteristics included nature of offense (according to the article of the Swiss Criminal Code), recidivism (yes/no reoffending), and multiple types of offenses (number of crime categories).

### Statistical analysis

Age, and number of different types of offenses were treated as continuous variables. Type of offense, recidivism, marital status (single / married), foreign nationality (yes / no), as well as social cognitive and emotional cognition (impairment yes/no) were treated as binary variables. Education (school drop-out / obligatory schooling / apprenticeship / high school, university) was treated as ordinal variables. Psychiatric diagnoses were coded according to ICD-10 codes. Criminal offenses were coded according to the Swiss Criminal Code. Cases with multiple offenses were considered in each diagnostic/offense category separately.

Group comparison between patients with and without SC impairment was analyzed with Fischer’s exact test for categorical variables, and with unpaired t-test for continuous variables. Multiple logistic regression models were applied to predict the presence of cognitive, respectively, emotional SC impairment. One participant was excluded from the cognitive SC regression models because of missing data. Independent predictors were demographic characteristics (age, nationality, level of formal education, marital status), criminal history (type of offense, number of convictions, number of different types of offense), and psychiatric diagnosis. Crimes were classified into interpersonal violent (crime categories a, d, e, f, i) and non-violent groups (c, g, h, j, k) as defined in Table 1. All statistics were performed using Stata release 18.5.

**Table 1:** Sample characteristics.

Two diagnostic groups	SSD^1^ (F20-F29)	PD^1^ (F60.2-F60.4)	Total
Demographics	*N* = 51 (68.0%)	*N* = 24 (32.0%)	*N* = 75 (100%)
Age mean (SD) (years)	34.1 (10.3)	31.9 (9.8)	33.4 (10.1)
Foreign nationality % (n)	29 (56.9%)	13 (54.2%)	42 (56.0%)
Marital status % (n)			
Single	46 (90.2%)	18 (75.0%)	64 (85.3%)
Divorced, widowed	5 (9.8%)	4 (16.7%)	9 (12%)
Married	0 (0.0%)	2 (8.3%)	2 (2.7%)
Education % (n)			
School drop-out	19 (37.3%)	7 (30.4%)	26 (35.1%)
Obligatory schooling	15 (29.4%)	10 (43.5%)	25 (33.8%)
Apprenticeship	14 (27.5%)	6 (26.1%)	20 (27%)
High school, university	3 (5.9%)	0 (0.0%)	3 (4%)
Intelligence quotient (IQ) % (n)			
IQ < 70	17 (33.3%)	5 (20.8%)	22 (29.3%)
IQ 70–80	15 (29.4%)	8 (33.3%)	23 (30.7%)
IQ > 80	19 (37.3%)	11 (45.8%)	30 (40.0%)
Criminal offenses (Swiss Penal Code) % (n)			
a. Physical violence (art. 111–136)	43 (84.3%)^2^	20 (87.0%)	63 (85.1%)
b. Drug trafficking (Lstup, art. 118–123)	30 (58.8%)	17 (73.9%)	47 (63.5%)
c. Property violation (art. 137–172)	32 (62.7%)	13 (56.5%)	45 (60.8%)
d. Violation forces of order (art. 285–295)	28 (54.9%)	13 (56.5%)	41 (55.4%)
e. Threat, sequestration (art. 180–186)	19 (37.3%)	14 (60.9%)	33 (44.6%)
f. Honor and privacy (art. 173–179)	16 (31.4%)	11 (47.8%)	27 (36.5%)
g. Road traffic laws (LCR, art. 90–99)	13 (25.5%)	8 (34.8%)	21 (28.4%)
h. Arson (art.221–230)	11 (21.6%)	4 (17.4%)	15 (20.3%)
i. Sexual offense (art. 187–200)	7 (13.7%)	7 (29.2%)	14 (18.6%)
j. Gun law violation (Larm)	9 (17.6%)	2 (8.7%)	11 (14.9%)
k. Illegal immigration (LEtr)	6 (11.8%)	2 (8.7%)	8 (10.6%)
Recidivism	39 (76.5%)	19 (79.2%)	58 (77.3%)
Multiple offenses (mean, SD, range)	4.5 (4.7) [1–9]	4.7 (3.9) [0–8]	4.6 (4.5) [1–9]

1 SSD, schizophrenia spectrum disorder; PD, Cluster B personality disorder.

2 Offense categories exceed 100% because patients committed multiple offenses.

## Results

### Sample characteristics

Sample characteristics are summarized in Table 1 according to two diagnostic groups: 51 SSD and 24 PD patients. Both groups are well balanced for demographic and criminal characteristics, as well as intelligence quotient and school education.

In both diagnostic groups, participants are male and aged early 30’s. There is no group difference for nationality (55% are of foreign nationality) and marital status (75–90% are single). Foreign nationality included origins from Africa 41%, West Europe 31%, Asia 15%, US 8% and East Europe 5%. Note that besides their foreign origins, patients had mostly grown up in Switzerland and are French speaking. They had been able to do neuropsychological assessment and receive intensive psychotherapeutic treatment in French language for several years. In Switzerland, inmates benefit from language classes during their prison stay when necessary.

Almost one third of the cases has dropped out from school because of conduct disorders, drug use or violent behavior. Another third has finished obligatory schooling and the later third has obtained an apprenticeship degree. Their general level of intelligence was low. About 40 % (37.3–45.8%) ranged in the lower average range (IQ > 80), one third was situated at the edge of the impairment range (IQ 70–80), and 20 to 30 % of the participants showed a clinically significant intellectual deficiency (IQ < 70). Note that the files excluded from the study included the same percentage of average IQ (42.8% IQ > 80) but a higher percentage of significant deficiency (50% IQ < 70).

Regarding criminal offenses, most of the inmates have been convicted for physical violence (bodily harm such as aggression, assault, fight, murder). Two-third were convicted for drug-related offenses and for violation of property (such as robbery, organized fraud, or breach of trust). Half of the sample have been convicted for violation against the forces of order. Less than half committed violation of domestic privacy (threats, sequestration, and kidnapping) or violation of honor and privacy. Violation of road traffic are reported in 25.5–34.8% and sexual offenses in 13.7–29.3% of the cases. Deliberately setting fire, illegal immigration and violation of gun law were the less frequent offenses in our sample. Most of the offenders relapsed into criminal behavior repeatedly and presented multiple types of crimes. All 75 participants were admitted for court-ordered treatment according to Swiss Criminal Code.

### Rates of social cognition impairment

Regarding cognitive SC, 54 patients passed the Intention inference task ToM cartoons (with a mean of 21.8 ± 5.0, range 10–28) and 20 patients the GeSOCS ToM cartoons (with a mean of 17.3 ± 3.2, range 6.5–20). Average IQ according to the diagnostic subgroup rates revealed to be similar for both task groups: IQ = 75.5 ± 14.3 and 69.2% SSD vs. 30.7% PD for Intention inference task, and IQ = 78.5 ± 14.2 and 63.1% SSD vs. 36.8% PD for the GeSOCS ToM task, excluding a potential systematic bias related to IQ based instrument selection. With respect to emotional SC, the mean score was 11.1 ± 2.1 (range 6–14.1). The excluded files presented the same profile of cognitive SC (mean of 17.7 ± 7.1, range 8–25) and emotional SC (mean score = 9.4 ± 3.6, range 6.3–14.1).

Regarding cognitive SC impairment, it was present in 39.2% (29/74) of the patients. In contrast, impairment in emotional SC was less frequent 25.6% (19/74). Table 2 explores SC according to our *a priori* hypothesis. With respect to crime recidivism, both cognitive and emotional SC impairments are present in 3/4 of recidivist patients and 1/4 of single offense patients. Considering the own-nationality advantage, impairment differs for emotional SC between foreigners (84%) and swiss (15%) nationality patients. This nationality-related difference is less salient for cognitive SC. Emotional as well as cognitive SC impairments are present in half of the patients with intelligence disability. In contrast, patients with average IQ present only low levels of impairment in cognitive SC (20%) and even lower in emotional SC (10%). According to diagnostic groups, patients with Cluster B personality disorders present the lowest level (10%) of emotional SC impairment, while SSD patients present high levels (82 and 89%) of cognitive and emotional SC impairment.

**Table 2:** Social cognition impairment rates.

Impairment	Cognitive SC (*N*=74^2^)		Emotional SC (*N* = 75)	
	Yes	No	Yes	No
N	29 (39.2%)	45 (60.8%)	19 (25.3%)	56 (74.7%)
Recidivism				
Single offense	7 (24.1%)	10 (22.2%)	4 (21.1%)	13 (23.2%)
Multiple offenses	22 (75.9%)	35 (77.8%)	15 (78.9%)	43 (76.8%)
Nationality				
Swiss	12 (41.4%)	20 (44.4%)	3 (15.8%)	30 (53.6%)
Foreign	17 (58.6%)	25 (55.6%)	16 (84.2%)	26 (46.4%)
Intelligence quotient (IQ)				
Disability IQ < 70	15 (51.7%)	7 (15.6%)	10 (52.6%)	12 (21.4%)
Edge IQ 70–80	8 (27.6%)	15 (33.3%)	7 (36.8%)	16 (28.6%)
Average IQ > 80	6 (20.7%)	23 (51.1%)	2 (10.5%)	28 (50.0%)
Diagnostic group^1^				
SSD	24 (82.8%)	27 (60.0%)	17 (89.5%)	34 (60.7%)
PD	5 (17.2%)	18 (40.0%)	2 (10.5%)	22 (39.3%)

1 SSD, schizophrenia spectrum disorder; PD, Cluster B personality disorder.

2 One participant presented missing data.

### Determinants of cognitive SC

Logistic regression models revealed that cognitive SC performance was positively predicted only by the intelligence quotient after adjusting for recidivism, nationality, and psychiatric diagnosis (Table 3). The model was statistically significant (*p* < 0.001) and explained 16% (pseudo-R^2^ = 0.16) of the variance of cognitive SC with a strong likelihood (*N* = 74, LR chi^2^(5) = 16.68). Despite the relatively low events-per-variable ratio for cognitive SC (EPV = 5), we privileged a model that fits our a priori hypotheses (instead of a random analysis that would imply correction for multiple comparisons), namely the impact of criminal recidivism, diagnostic group, and intelligence quotient on cognitive SC, after adjustment for nationality. Higher levels of cognitive SC are predicted by higher overall cognitive skills only, independently of the psychiatric diagnosis group and criminal recidivism. Intelligence clearly acts as a protective factor against cognitive SC impairment and this protective effect doubles from 4 to 9-fold for normal range intelligence (>80).

**Table 3:** Predictors of cognitive SC in multiple regression model.

Cognitive SC (1 = intact)	Odds ratio	*p*	[95% CI]
Recidivism (yes^1^)	1.35	0.646	[0.37, 4.86]
Nationality (foreign)	1.62	0.412	[0.51, 5.15]
Intelligence quotient (IQ < 70)			
Edge IQ 70–80	**4.45**	0.030*	[1.16, 17.11]
Average IQ > 80	**9.97**	0.001**	[2.42, 41.09]
Diagnostic group (SDD)			
PD	2.94	0.081	[0.87, 9.92]

Model significance: *N* = 74, EPV = 5, LR chi^2^(5) = 16.68, pseudo-R^2^ = 0.16, *p* < 0.001. *p < 0.05, **p < 0.01.

1 Reference categories: yes (recidivism), foreign (nationality), deficiency IQ<70 (intelligence quotient), schizophrenia spectrum disorder SDD (diagnosis) vs. Cluster B personality disorder PD. Significant values are highlighted in bold.

### Determinants of emotional SC

Logistic regression models showed that emotional SC performance was increased by average intelligence quotient and PD diagnostic group, and decreased by foreign nationality, after adjusting for recidivism (Table 4). The model was statistically significant (*p* < 0.001) and explained a higher percentage variance of emotional SC compared to cognitive SC, namely 28% (pseudo-R^2^ = 0.28), with a strong likelihood ratio (*N* = 74, LR chi^2^(5) = 23.89). For the sake of comparison with cognitive SC, and despite the low events-per-variable ratio for affective SC (EPV = 3.8), we present the regression model with the same predictors. While healthy intelligence and personality disorder act as protective factors compared to SSD and intellectual deficiency, foreign nationality acts as a risk factor, increasing the odds of an emotional SC impairment by 84%. Of importance, recidivism is not significantly related to their level of cognitive and emotional SC.

**Table 4:** Predictors of emotional SC in multiple regression model.

Emotional SC (1 = intact)	Odds ratio	*p*	[95% CI]
Recidivism (yes^1^)	0.68	0.615	[0.15, 3.05]
Nationality (foreign)	**0.16**	0.016*	[0.04, 0.71]
Intelligence quotient (IQ < 70)			
Edge IQ 70–80	1.24	0.792	[0.29, 5.04]
Average IQ > 80	**8.83**	0.016*	[1.49, 52.08]
Diagnostic group (SSD)			
PD	**7.92**	0.018*	[1.42, 44.29]

Model significance: *N* = 74, EPV = 3.8, LR chi^2^(5) = 23.89, pseudo-R^2^ = 0.28, *p* < 0.001. *p < 0.05, **p < 0.01.

1 Reference categories: multiple offenses (recidivism), foreign (nationality), deficiency IQ<70 (intelligence quotient), schizophrenia spectrum disorder SDD (diagnosis) vs. Cluster B personality disorder PD. Significant values are highlighted in bold.

## Discussion

### Main findings

This study aimed to assess in the determinants of cognitive and emotional SC abilities in an adult male sample of forensic psychiatric offenders, according to their psychiatric diagnosis subgroup (schizophrenia spectrum disorder vs. Cluster B personality disorder), level of intelligence quotient, and history of violent or non-violent crime. Our data show that 25% of the total sample performed below the clinically significant cut-off score in an emotional SC (facial emotion recognition) and 39% in a cognitive SC task (theory of mind comics). Cognitive SC impairment was related to lower intelligence quotient (IQ) only, while the risk for emotional SC impairment was increased in cases with lower IQ and schizophrenia spectrum diagnosis (as opposed to PD). Interestingly, no differences emerged according to the criminal recidivism.

In respect to cognitive SC, the present findings are consistent with early contributions in this field. Brüne (2003) showed that the lower ToM performances observed in schizophrenics compared to controls did not persist after controlling for IQ (Brüne, 2003). Murphy (2006) also found that schizophrenia patients perform worse in ToM tasks compared to patients with personality disorders. This difference was explained by the higher levels of general intellectual functioning observed in this latter group. The present data suggest that patients with average IQ (> 80) have substantially higher chances to be protected from cognitive SC impairment (OR = 9.97, 95% CI: 2.42–41.09). However, the wide confidence interval indicates that while the protective effect appears robust, the precise magnitude remains uncertain and could range from approximately 2.5-fold to over 40-fold protection. This probability drops to 4-fold for patients within the edge IQ range (70–80), independently of their diagnostic group. Results confirm our hypothesis of a strong relationship between general intelligence and social cognition, but only for the cognitive SC. Importantly, our results in a forensic sample of patients with court-ordered treatments are consistent with those reported in the general population and in children with intellectual disabilities (Thirion-Marissiaux and Nader-Grosbois, 2008; Conte et al., 2018a), indicating that the level of general intelligence and not the psychiatric diagnosis is the main parameter to consider when assessing the cognitive SC abilities. To have realistic expectations in psychotherapeutic settings, mental health professionals would greatly benefit by assessing systematically patients’ IQ before investing to a treatment of social abilities aiming to favor interpersonal prosocial behaviors.

A different pattern emerges when studying the predictors of emotional SC, as assessed with a facial emotion recognition task. Only one quarter of the sample shows emotional SC impairment. This ability was better preserved in patients with PD, average IQ and swiss nationality patients, independently of patients’ history of crime recidivism. Regarding cultural differences, due to an own-nationality advantage, individuals score better at recognizing facial expressions of basic emotions posed by individuals from their country than those of other-nationality members (Yan et al., 2016). Culture - as an intricate system of social concepts and beliefs - finely shapes the internal representations of common facial expressions of emotion, generating different expectations of facial expressions signals (Jack et al., 2012). The interpretation of these results needs caution because we cannot exclude a bias due to the assessment material. The Western-centric pictures of the task lead to an underrepresentation of African and Asian populations and may have amplified the higher accuracy for same-culture faces or caused confusion between emotions that are culturally subtle. Further, to a lesser extent than in cognitive SC, our data suggest that average range IQ is an additional protective factor for emotional SC (OR = 8.83, 95% CI: 1.49–52.08). The very wide confidence interval reflects substantial uncertainty in the effect magnitude, warranting cautious interpretation of this finding.

Regarding diagnosis, our results confirm the well-known facial emotion recognition deficit in schizophrenia patients, both violent and non-violent, supporting the idea that it represents a trait feature of the illness (Demirbuga et al., 2013; Bordon et al., 2017; Tasios et al., 2024). Schizophrenia patients report significantly more negative emotional contagion, overwhelming emotions, and symbolization of emotions by imagination, and less self-control of emotional expression than healthy persons (Lehmann et al., 2014). In contrast, patients with antisocial and borderline personality disorders are less impaired in emotional SC, compared to schizophrenics. Unlike schizophrenia, patients with antisocial personality disorders show specific deficits in the recognition of aversive cues in others and in particular fearful expressions (Decety and Moriguchi, 2007; Marsh and Blair, 2008). In offenders, externalizing was associated with faster processing for angry faces under greater ambiguity in the absence of contextual threat (Brennan and Baskin-Sommers, 2021). One possible explanation for this diagnosis-related difference may be that antisocial offenders display only a hostile attribution bias, in that they rate ambiguous fear-anger expressions as angrier, with a lower threshold to detect anger compared to the general population. This effect has been reported to be more pronounced for male faces and correlates with self-reported aggression, in the absence of a consistent deficit in recognizing other types of emotions (Wegrzyn et al., 2017).

Unexpectedly, and despite the hostile attribution bias hypothesis, we found no direct relationship between SC impairment and the patients’ recurrency of violent behavior. Our results show that neither emotional nor cognitive SC impairments are related to offense behavior, not all offenders show SC impairments. Previous evidence had found a consistent relationship between violent behavior and emotional SC impairment. Conflicting data were obtained for cognitive SC impairment (Winter et al., 2017; Friestad and Vaskinn, 2021; Tasios et al., 2024). These discrepancies may be explained by the differential adjustment for general intelligence. Neither Friestad and Vaskinn (2021), nor Tasios et al. (2024), have included IQ assessment in their study. Winter et al. (2017) included IQ as covariate, with an average offender IQ of 95 (±12), that was situated above the present study. In our multivariate regression models including both criminal recidivism and IQ, recidivism has not emerged as a significant predictor. These findings need be further explored in future studies with larger samples of mentally disordered offenders, using distinctive cognitive and emotional SC assessments, as well as IQ and history of substance abuse as cofounding variables.

### Strengths and limitations

Strengths of the present study include the homogeneity of the clinical sample. Both patient groups were treated in the same forensic clinic and followed the same court-ordered psychiatric treatments. Psychiatric diagnoses were confirmed by two different senior forensic psychiatrists and rated according to ICD-10 standards and criteria. Social cognition was assessed by observer ratings performed by the same experienced forensic psychiatric neuropsychologist in all 75 patients, once patients were clinically stable.

However, several limitations need to be considered. First, since our data were extracted from clinical files in this prospective study design, cognitive SC data was assessed with two different theory of mind instruments, as is frequent in routine clinical settings. Indeed, there is yet no general test that allows neuropsychologists to assess ToM and social cognition function in everyday clinical practice. There are no standardized Theory of Mind tests that are adaptable to different cognitive profiles, such as adult individuals with language poverty, and intellectual or memory impairments. Non-verbal intentions attribution comic strip tests are considered the strongest psychometric option in clinical and forensic settings (Le Donne et al., 2023). Nevertheless, in adult populations, ceiling effects are frequent, interrelations among measures remain poor, and current reviews highlight a large evidence gap (Yeung et al., 2024). To allow for comparison, continuous scores of both scales were merged using the clinical cut-offs and transformed into a binary variable. Although both scales are of the same nature, the use of one identical scale would have allowed to use a more informative continuous variable. Second, SC assessment is limited by cultural bias and lacks validation of culturally sensitive tools (Ziermans et al., 2026). We choose to use visual material (pictures and cartoons) to limit language barriers, using measures that have been validated in French speaking populations. Third, psychiatric diagnoses were rated by two independent psychiatrists, yet without standardized structured interviews. We are thus not able to ascertain inter-reliability statistics for both raters. Fourth, forensic psychiatry patients frequently present comorbid substance use disorders that may affect SC. The relationship between SC impairment and violence may be mediated by substance use in SSD as well as in PD patients (Preller et al., 2014; Uhlmann et al., 2016; Karabulut and Uçkun, 2025). Although patients in this study were abstinent at the time of the data assessment and living in the drug-free environment of a forensic clinic, their past drug use was not systematically assessed. 70% of patients with substance use disorders show social cognition impairment in facial affect recognition but not in ToM, compared to healthy controls (Pinon-Blanco et. 2025). We cannot exclude that past comorbid substance use has produced additional IQ and social cognition impairments independently of patients’ current abstinence status. Further studies are needed to explore the joint impact of SDD or PD with and without history of substance use disorders. In the same line, no control was made for the type and doses of psychotropic medication as well as symptom severity, that may impact on the SC assessment. However, this latter bias has been indirectly considered since the well-trained clinical neuropsychologist (10 years of experience with forensic inpatients) who has performed all assessments checked on the optimal clinical stability before administering the tests. Finally, sample size is relatively limited, with low events-per-variable ratios, leading notably to large confidence intervals for Average IQ and PD diagnostic group predictors, especially for emotional SC. Effect size and the model-fit statistics of the logistic regression models confirmed their stability. However, the wide confidence intervals imply limitations in the degree of statistical certainty imposing additional caution in the interpretation of our findings. In the light of this observation, our results should be considered as preliminary evidence and need to be confirmed in a larger population sample. The present study focused on SSD and PD related emotional and cognitive SC differences. Future studies are warranted to control for PD subtype-specific SC impairments.

### Implications

This study, addressing social cognition in 75 adult forensic psychiatry patients showed that cognitive and emotional SC are determined by different predictors. Both PD and SSD patients, with IQ scores > 70, may benefit from cognitive SC training in forensic mental health settings. Patients with PD and average range IQ scores (>80) may benefit from emotional social cognition training. To determine PD subtype-specific SC training objectives, assessments need to be individualized. Indeed, future studies in larger samples are needed to explore potentially heterogenous patterns of emotional SC impairment according to PD subtype, e.g., antisocial PD show specific fear recognition deficits while borderline PD show hypersensitivity to negative emotional cues.

In clinical practice, referral for social cognition training should depend on patients’ level of intelligence and include differential emotional and cognitive SC baseline assessment. The rate of intellectual deficiency (29% IQ < 70) in this sample is above the rate (14%) generally diagnosed in our local forensic population (Weber et al., 2023). Plus, 50% of the patients excluded from the present study sample because of lacking SC assessment, presented with intellectual deficiency. It is thus likely that low IQ associated SC impairments are possibly underestimated in our findings and their prevalence needs to be replicated in a larger forensic population. Our data reveal that low IQ forensic psychiatric patients do not benefit from routine standardized SC assessment. Forensic legal authorities need to consider patients’ level of intelligence when ordering behavioral therapies aiming to improve patients’ prosocial abilities.

## Data Availability

The original contributions presented in the study are included in the article material, further inquiries can be directed to the corresponding author.

## In memoriam

Dedicated to the memory of FH.
